# Thyroid computed tomography imaging: pictorial review of variable pathologies

**DOI:** 10.1007/s13244-016-0506-5

**Published:** 2016-06-07

**Authors:** Mnahi Bin Saeedan, Ibtisam Musallam Aljohani, Ayman Omar Khushaim, Salwa Qasim Bukhari, Salahudin Tayeb Elnaas

**Affiliations:** Department of Radiology, MBC-28, King Faisal Specialist Hospital and Research Center, P.O Box 3354, Riyadh, 11211 Saudi Arabia; Medical Imaging Department, Ministry of National Guard, Health Affairs, King Abdulaziz Medical City, Riyadh, Saudi Arabia; Department of Radiology, University of Tabuk, Tabuk, Saudi Arabia

**Keywords:** Incidental thyroid nodule, Thyroid cancer, Goiter, Ectopic thyroid, Computed tomography

## Abstract

**Abstract:**

Focal and diffuse thyroid abnormalities are commonly encountered during the interpretation of computed tomography (CT) exams performed for various clinical purposes. These findings can often lead to a diagnostic dilemma, as the CT reflects the nonspecific appearances. Ultrasound (US) examination has a superior spatial resolution and is considered the modality of choice for thyroid evaluation. Nevertheless, CT detects incidental thyroid nodules (ITNs) and plays an important role in the evaluation of thyroid cancer.

In this pictorial review, we cover a wide spectrum of common and uncommon, incidental and non-incidental thyroid findings from CT scans. We also discuss the most common incidental thyroid findings, best practices for their evaluation, and recommendations for their management. In addition, we explore the role of imaging in the assessment of thyroid carcinoma (before and after treatment) and preoperative thyroid goiter, as well as localization of ectopic and congenital thyroid tissue.

***Teaching Points*:**

*• Thyroid disorders tend to have non-specific CT appearances*.

*• ITNs are common on neck CT*.

*• ITN management depends on nodule size, age, health status, lymphadenopathy, and invasion*.

*• CT is used in assessment of cancer extension, mass effect, invasion, and recurrence*.

*• CT plays a role in preoperative planning in patients with symptomatic goiter*.

## Introduction

Thyroid disorders are common and include many entities. They can be symptomatic, asymptomatic, diffuse, focal, neoplastic, or non-neoplastic processes. Neck ultrasound (US), with the prospect of proceeding to fine needle aspiration (FNA), is the first line of investigation; however, other options are available. Thyroid Uptake Scans using Tc-99 m or I-123 are typically reserved for specific clinical scenarios. Cross-sectional imaging including computed tomography (CT) and magnetic resonance imaging (MRI) detect incidental thyroid nodules (ITNs) and can be used in the evaluation of thyroid cancers and goiter [1, 2]. The aim of this article is to provide a pictorial review of a broad spectrum of incidental and non-incidental thyroid findings on CT scans.

## CT scans of the thyroid: normal anatomy and imaging techniques

The thyroid gland is a vascular, encapsulated structure made up of right and left lobes, which are connected at the midline by the isthmus. Each lobe is about 2 cm thick, 3 cm wide, and 5 cm long. The thyroid apex is located superiorly at the level of the mid-thyroid cartilage. The inferior margin of the gland is at the level of the fifth or sixth tracheal ring. The thyroid gland is encapsulated by the middle layer of deep cervical fascia and is part of the visceral space in the infrahyoid neck. It wraps around the trachea and is separated from the oesophagus by the tracheoesophageal groove on each side, which houses the recurrent laryngeal nerves. The thyroid has variable lymphatic drainage to the internal jugular chain, para-tracheal region, mediastinum, and retropharyngeal area. It has homogeneous high attenuation values on a CT scan, as compared to adjacent muscles, due to its high iodine concentration. It shows avid iodine contrast enhancement due to its hypervascularity [1, 3].

Multi-detector volumetric acquisition from the skull base to the tracheal bifurcation is usually obtained. Multiplanar 2-mm axial, coronal, and sagittal images are typically available. Examination can be acquired with or without administration of intravenous (IV) iodinated contrast.

## Incidental thyroid findings on CT scan

The thyroid gland can have variable CT scan findings, such as calcifications, single or multiple nodules, cysts, or diffuse enlargement.

**Fig. 1:**
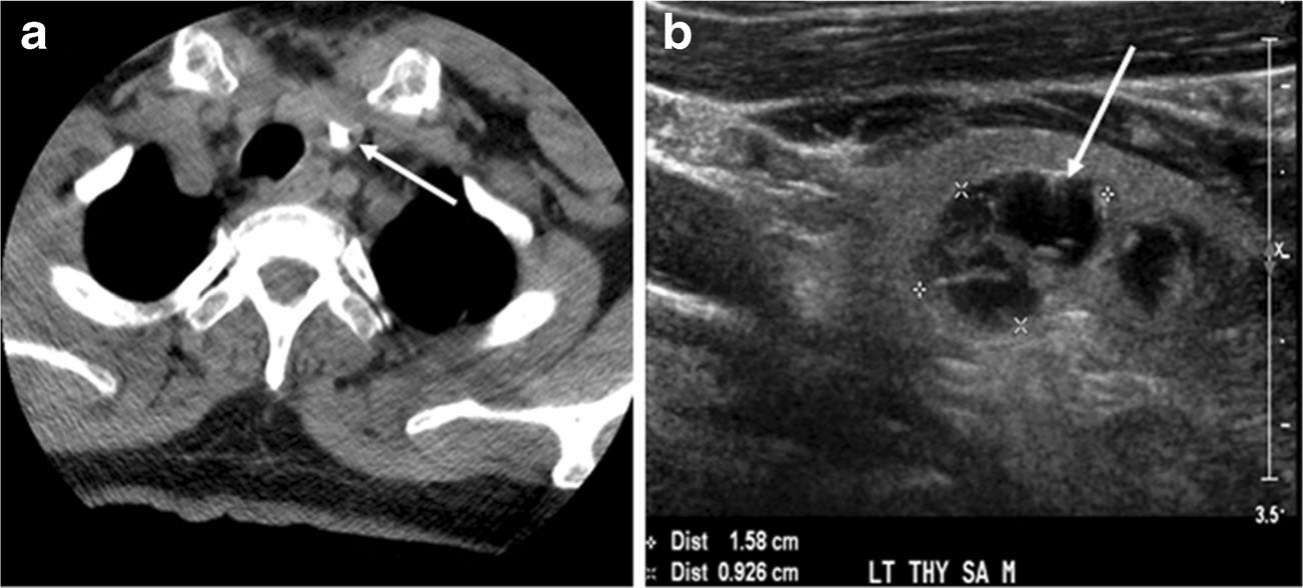
An incidentally discovered colloid nodule with calcification, shown on CT scan of a 58-year-old female patient. **a** Non-enhanced axial CT scan of the neck demonstrates a coarse calcification at the left thyroid inferior pole. **b** Sagittal grey scale ultrasound of the thyroid demonstrates a heterogeneous nodule with predominant cystic component. Calcification was not seen in the ultrasound, probably due to its lower location in the superior mediastinum

**Fig. 2:**
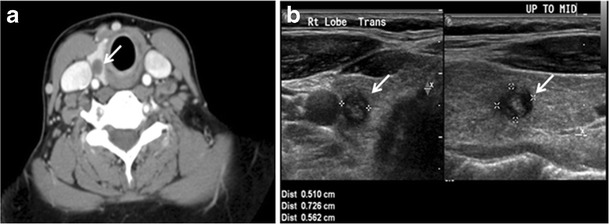
A 51-year-old female patient post left hemi-thyroidectomy, with incidentally discovered right thyroid colloid nodule on CT scan. **a** Enhanced axial CT scan of the neck demonstrates a well-defined, hypodense right thyroid nodule (*white arrow*) with no internal calcifications or cervical lymphadenopathy. **b** Transverse greyscale thyroid ultrasound demonstrates a well-defined, hypoechoic right thyroid lobe nodule with a central echogenicity including comet tail (ring down) artefacts (*white arrow*). No vascularity (not shown) or calcifications were detected

Thyroid calcifications on a CT scan can be seen in both benign and malignant thyroid lesions [1]. Sonographic examination of the thyroid can differentiate between micro-calcifications, which are highly associated with papillary thyroid carcinoma, and eggshell calcifications, which favour a benign process such as colloid cysts (Figs. 1 and 2) [4]. In a retrospective review of preoperative CT scan, 35 % (135 of 383) of the patients had detectable intra-thyroidal calcifications. Among them, 48 % had a histopathologically proven thyroid cancer. Calcified nodules had a significantly higher incidence of thyroid cancer and lymph node metastases. The incidence of thyroid cancer among nodules with different calcifications patterns were 79 % of nodules with multiple punctate calcifications, 58 % of nodules with a single punctate calcification, 21 % of nodules with coarse calcification, and 22 % of nodules with peripheral calcification. Most of the single calcified nodules were malignant [5]. However, this did not include patients with ITNs and the sample is skewed towards malignancy. Another study evaluated the presence of ITNs on CT scans and found that 12 % of thyroid nodules were calcified, with no significant correlation between malignant or potentially malignant histology and punctate calcifications [6]. As a result, some researchers believe that calcification per se is not a suspicious CT sign, and have suggested that calcified thyroid nodules on CT scans should be treated the same as non-calcified nodules [7].

Thyroid cystic changes are variable, ranging from simple cysts with a thin wall to complex cysts with septations and solid components. An adenoma may undergo cystic degeneration. It is important to note that papillary carcinoma may mimic a benign-looking cyst. Simple serous cysts appear with fluid density on a CT scan, whereas a cyst with haemorrhage or high thyroglobulin content is iso-dense to muscle [1].

**Fig. 3:**
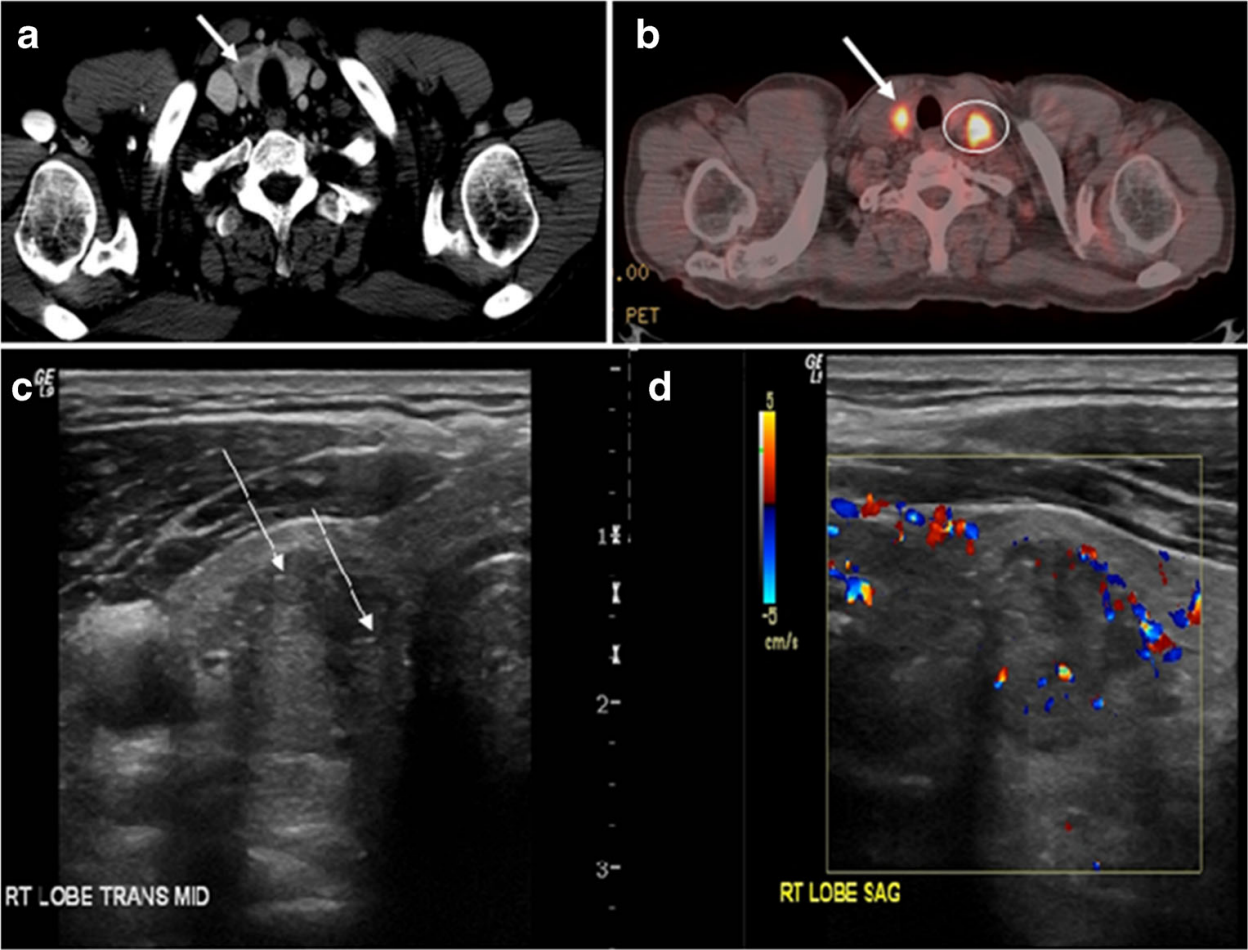
An incidental PTC in a 62-year-old male patient with lymphoma. **a**, **b** Enhanced axial CT scan and fused PET/CT scan of the neck demonstrate a well-defined, hypodense right thyroid nodule (*white arrow*) with high FDG uptake. The FDG-avid uptake in the left side (*circle*) is related to patient’s known lymphoma, which resolved after treatment. **c**, **d** Transverse greyscale and sagittal colour Doppler ultrasound of the neck demonstrate a right thyroid irregular hypoechoic lesion with some micro-calcifications (*white arrows*) and increased vascularity

Thyroid nodules that are detected by an imaging study but have not been previously detected or suspected clinically are considered to be ITNs. ITNs are one of the most common incidental findings on neck imaging. ITNs are reported in up to 25 % of chest CT scans, and in 16–18 % of cervical region cross-sectional imaging, including CT and MRI scans. The rate of malignancy in the detected ITNs on CT and MRI scans varies from 0 % to 11 % [6, 8–10]. Incidentally detected thyroid carcinomas are more likely to be papillary thyroid carcinomas (PTCs) (Fig. 3). Incidentally detected cancers tend to be smaller in size and less likely to have distant metastasis, as compared to clinically suspected thyroid cancers [11].

On CT scans, a malignant lesion is suspected when the margins are ill-defined and there is extra-thyroid extension, lymph node involvement, or invasion of the surrounding structures. The absence of these features does not exclude malignant tumours, especially papillary, follicular, and medullary thyroid carcinomas (Fig. 3) [1]. Therefore, ultrasound is the modality of choice for thyroid lesion evaluation, due to its superior spatial resolution compared to CT examinations. Sonographic features of malignancy are micro-calcifications, acoustic shadowing, anti-parallel orientation, marked hypoechogenicity, irregular or microlobulated margins, and increased vascularity. CT scans lack the ability to detect these reliable sonographic signs of malignancy. Therefore, further management of ITNs, if required, usually begins with thyroid ultrasound and FNA should be considered according to the ultrasound findings [4, 12–15].

The American College of Radiology (ACR) flowchart and recommendations for ITNs detected by CT or MRI offer general guidance and are not applicable to all patients. The recommendations are primarily based on the presence or absence of suspicious features, nodule size, patient’s age, patient’s life expectancy, and patient’s comorbidities [11]. Suspicious features that can be detected on CT scans include signs of local invasion and abnormal lymph nodes. Abnormal lymph nodes may show cystic components, calcifications, and/or increased enhancement. Mere nodal enlargement is less specific for thyroid cancer metastasis; however, further evaluation should be considered if the ITN has ipsilateral jugulo-digastric lymph nodes > 1.5 cm on the short axis or > 1 cm for other groups. Cervical Level IV and VI lymphadenopathies raise a higher suspicion of thyroid carcinoma metastasis. Almost all patients with ITNs and suspicious imaging features should be evaluated with a neck ultrasound. Patients with comorbidities or limited life expectancy without suspicious features should not undergo further evaluation. Nevertheless, further workup might be necessary for such individuals if it is clinically warranted, or specifically requested by the referring physician or the patient [11].

In patients with ITNs, it is important to inquire about pertinent historical factors predicting malignancy. These factors include history of childhood or adolescent head and neck or total body radiation exposure, and familial thyroid carcinoma or thyroid cancer syndrome. Syndromes associated with thyroid cancer include multiple endocrine neoplasia 2, familial adenomatous polyposis, Carney complex, Cowden’s disease, and Werner syndrome/progeria. If a patient has a first-degree relative with such a syndrome, screening based on the various components of that syndrome is advised. Nevertheless, there are no guidelines specifically addressing ITNs detected on CT scans in patients at risk of thyroid cancer. Therefore, in the absence of suspicious features on the CT scan, other criteria such as nodule size on the CT scan, patient age, and levels of thyroid stimulating hormone (TSH) are important in guiding management in such a patient population [16].

Although the correlation between thyroid nodule size and malignancy risk is limited, nodule size affects prognosis in malignant nodules [13]. Small thyroid cancers (less than 2 cm) tend to have an indolent course, with favourable prognosis even if not treated [9, 17]. Less than 7 % of the imaging-detected ITNs are seen in younger populations [6, 9, 18]. However, Shetty et al. [6] found a higher rate of malignancy in the ITNs detected on CT scans among patients younger than 35 years. Ito et al. [19] found a higher tumour progression risk among young patients (<40 years) with subclinical, low-risk PTCs who undergo observation rather than surgery. Therefore, nodule size and patient age should determine the need for workup in the general population without suspicious imaging features and with normal life expectancy. Further evaluation with ultrasound is required for patients less than 35 years old with nodules measuring more than 1 cm in the axial plane. The cutoff size for further evaluation is raised to 1.5 cm for patients more than 35 years old. This recommendation should be applied to the largest thyroid nodule in cases of multiple thyroid nodules. Incidentally discovered heterogeneous and enlarged thyroid glands should undergo dedicated ultrasound if the patient has no limited life expectancy or serious comorbidities [11].

## Thyroid cancers

### Epidemiology

Primary thyroid carcinomas include papillary, follicular, medullary, and anaplastic carcinomas. Lymphoma and metastasis of other primary malignancies to the thyroid gland represent a minority of thyroid carcinomas. Differentiated thyroid carcinomas (DTCs) originate from follicular epithelial cells and encompass PTCs and follicular thyroid carcinomas, including the Hurthle cell variant of follicular carcinoma. DTCs have an excellent prognosis and fortunately represent the majority of thyroid carcinomas. PTCs and follicular thyroid carcinomas represent 88 % and 8 %, respectively, of all thyroid malignancies. Medullary thyroid carcinoma arises from neuroendocrine C-cells and has a good prognosis. Anaplastic carcinoma is an aggressive undifferentiated tumour that usually affects the elderly and tends to have a worse prognosis [1, 2, 20, 21].

### Role of imaging

**Fig. 4:**
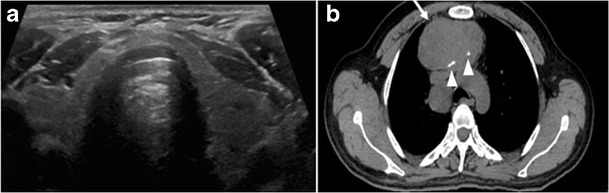
A 45-year-old male patient presented with anterior mediastinal metastatic PTC lesions and occult primary on imaging. Histopathology examination of the resected thyroid gland revealed micro-foci of PTC; the largest, in the isthmus, measured 4 mm. **a** Transverse greyscale ultrasound of the thyroid demonstrates homogenous gland with normal echogenicity and size. No focal lesion or micro-calcifications. **b** Non-enhanced CT scan obtained as part of PET/CT examination shows a heterogeneous, large, relatively dense anterior mediastinal mass (*white arrow*) with peripheral calcification (arrowheads). Thyroid gland has normal CT appearance with no abnormal FDG uptake (not shown)

**Fig. 5:**
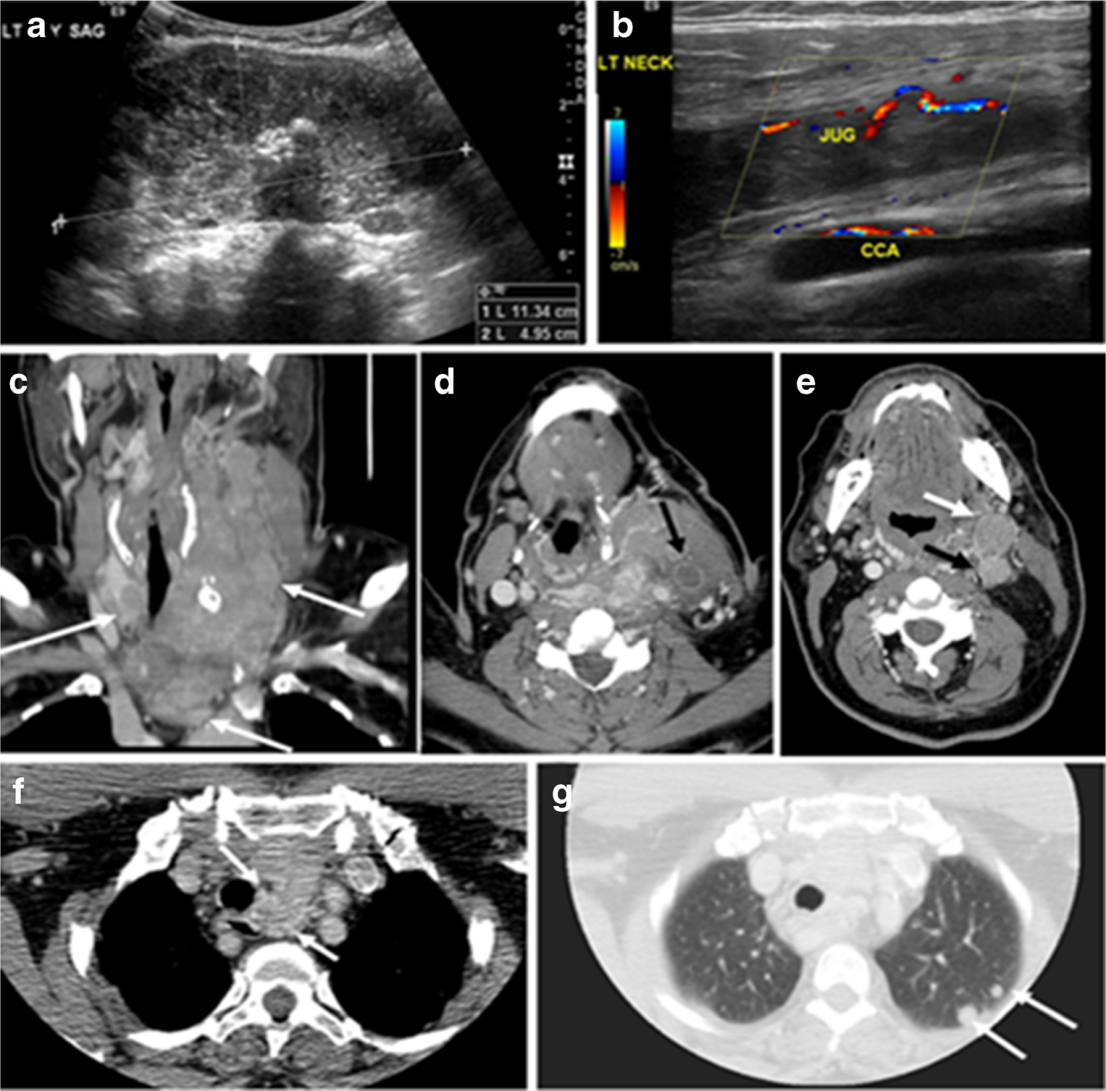
A poorly differentiated invasive left thyroid mass in a 58-year-old female patient. **a** Sagittal greyscale neck ultrasound shows a large hypoechoic lesion with macro-calcification and micro-calcification. **b** Sagittal colour Doppler ultrasound shows left internal jugular vein filling defect with detected internal vascularity suggestive of tumour thrombus. **c** Enhanced axial and coronal CT scans of the neck show heterogeneously enhancing large lesion replacing the left thyroid lobe and extending to the isthmus and the medial aspect of the right thyroid lobe (*white arrow*). The mass and the conglomerate lymph nodes measure 12.5 × 7 × 5.8 cm (*white arrows*). **d**, **e** Axial enhanced CT scans show enlarged left cervical nodes (white arrow) and left internal jugular vein (IJV) thrombus (black arrows). Note the IJV distention and central enhancing portion in the upper cut (*black arrow* in **e**) concerning the tumour thrombus. **f**, **g** Enhanced axial CT scan of the upper chest demonstrate a mass extension into the retrosternal area, left tracheoesophageal groove, and posterior to the trachea (*white arrows* in **f**). There are multiple bilateral pulmonary nodules (*white arrows* in **g**)

**Fig. 6:**
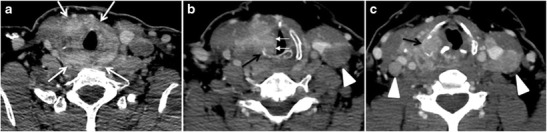
A 61-year-old female patient with locally aggressive PTC. **a** Enhanced axial CT scan of the neck demonstrates a heterogeneous infiltrative thyroid mass. This mass diffusely involves the entire gland and circumferentially encases the trachea with involvement of bilateral tracheoesophageal grooves (*white arrows*). **b**, **c** Additional axial cranial images show right cricoid cartilage destruction (*black arrows* in **b**), right thyroid cartilage destruction (*black arrow* in **c**), right vocal cord paralysis (*white arrows* in **b**), and bilateral cervical lymphadenopathy (*arrowheads*)

Surgery is the primary mode of treatment for DTCs. Post total thyroidectomy radioactive iodine (RAI) ablation is an option, especially in patients with distant metastasis, tumours larger than 4 cm, or extra-thyroidal disease extension. Ultrasound examination is usually adequate in evaluating primary tumours and cervical lymph nodes. Preoperative cross-sectional imaging with CT or MRI is indicated if there is a concern for local invasion that may alter the patient’s staging as well as surgical approach (Figs. 4, 5 and 6) [2]. Some thyroid primaries may be small, diffuse, or multifocal and therefore may be occult on imaging (Fig. 4) [6].

In patients with known thyroid malignancies, a non-enhanced exam is preferred due to the possible undesired interference of free iodide contrast medium with thyroid iodide I-131 uptake for 6–8 weeks or more [22, 23]. This would adversely affect the management of these patients by delaying diagnostic thyroid scintigraphy and radioiodine ablation in patients with DTCs for 2–6 months.

The radiologist must evaluate the central structures draping the thyroid gland including the trachea, oesophagus, larynx, and pharynx, as well as the recurrent laryngeal nerve. Invasion is suspected if the thyroid mass abuts the airway or oesophagus for more than 180 degrees. Luminal deformity, mucosal thickening, and mucosal focal irregularity are more specific indicators of invasion. Obliteration of the fat planes of the tracheoesophageal groove in three axial images and signs of vocal cord paralysis are indicative of recurrent laryngeal nerve invasion. Invasion of these central structures meets the criteria for T4a disease (Figs. 5 and 6) [2].

Arterial invasion constitutes T4b disease, which may preclude curative surgery. More than 180 degrees of arterial encasement is suggestive of invasion, however, arterial deformity or narrowing is much more suspicious for invasion [2]. The carotid artery is the most commonly involved artery; however, the mediastinal vessels should also be examined. Encasement of the carotid artery or mediastinal vessels for more than 270 degrees is unlikely to be resectable [24]. On the other hand, occlusion or effacement of the internal jugular vein can occur without invasion and does not influence surgical resectability or staging. Asymmetry of the strap muscle and the tumour abutting its external surface are signs of invasion. However, invasion of the pre-vertebral musculature is more challenging, as a large lesion can compress the muscle without invasion (Figs. 5 and 6) [2, 25].

Finally, the possibility of metastatic disease should be excluded. PTCs and medullary thyroid carcinomas tend to metastasize to regional lymph nodes. According to the AJCC/UICC TNM staging system, the nodal stage is classified by site: N1a indicates level VI nodal involvement, including paratracheal nodes; N1b indicates unilateral or bilateral lateral cervical nodal disease or superior mediastinal nodal disease (Figs. 4, 5 and 6) [2, 25].

The incidence of hematogenous spread of follicular carcinomas is 21–33 % and that of PTCs is 2–14 % [26, 27]. In medullary thyroid cancer and anaplastic thyroid cancer, distant metastasis was reported in 25 % and 40 % of patients, respectively. Distant metastases from DTCs tend to have a more favourable prognosis. Distant metastatic disease may appear years after the initial presentation. Therefore, imaging for distant metastases is usually done pre-operatively for anaplastic thyroid cancer and post-operatively for DTCs. DTC distant metastases sites include the lung (50 %), bone (25 %), lung and bone (20 %), followed by other sites (5 %) [28–30].

### Recurrence

The thyroid cancer recurrence rate is reported to range from 7 % to 14 % [31, 32]. Recurrence is usually detected within the first decade after initial disease diagnosis. Large lymph node metastasis is considered the strongest predictor for thyroid cancer recurrence [31]. Post-treatment surveillance for recurrent disease depends on cancer type and staging. Patients with DTC are usually treated with total thyroidectomy and RAI ablation. Patients should have baseline neck US evaluation at 6–12 months after the RAI ablation and then periodically, depending on the patient’s risk for recurrent disease and thyroglobulin (Tg) status. After the first post-operative RAI ablation, further RAI imaging is not necessary if the patient has normal neck US, undetectable Tg level under TSH stimulation, and negative antithyroglobulin (TgAb). Annual neck US with or without FNA, along with measurement of serum Tg and serum TgAb, is usually sufficient for post-treatment surveillance in those patients. Moreover, annual US is appropriate in patients with medullary cancer and normal calcitonin levels [2, 16, 33].

**Fig. 7:**
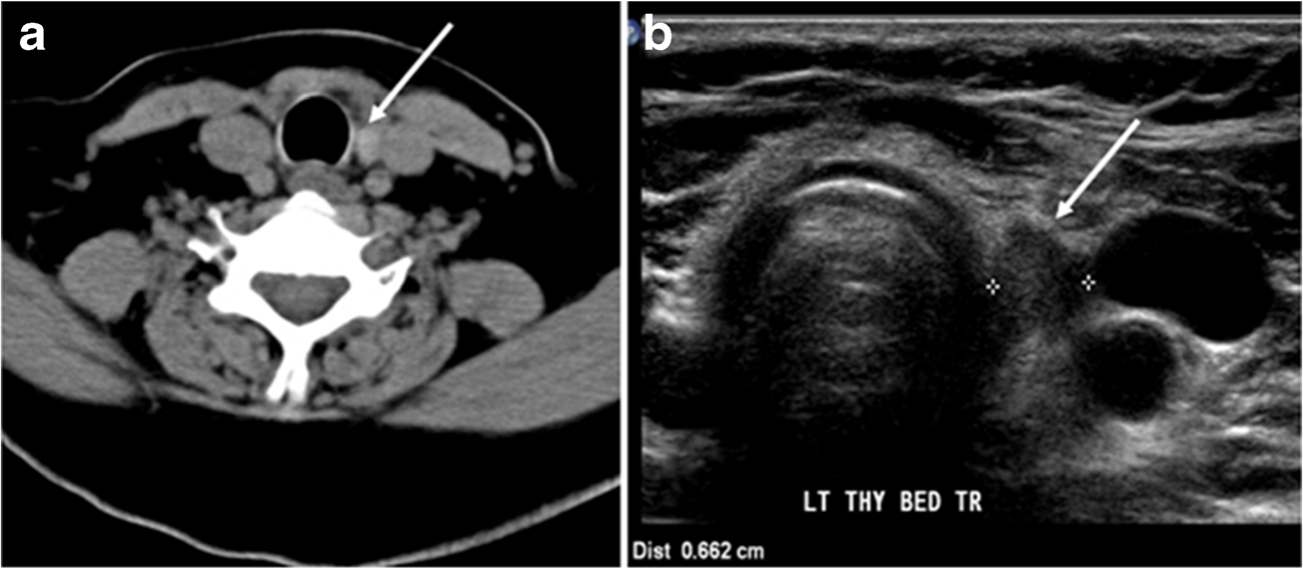
A 51-year-old female patient post total thyroidectomy for PTC with elevated thyroglobulin measurement. **a** Axial non-enhanced CT scan of the neck at the level of the thyroid bed demonstrates a well-defined, rounded, homogenously dense soft tissue situated between the trachea and left internal jugular vein (*white arrow*). **b** Transverse ultrasound image of the neck demonstrates a well-defined, homogenous, hypoechoic soft tissue nodule measuring 6 mm (*white arrow*) with no detected micro-calcifications. Biopsy showed a predominantly residual normal thyroid tissue with micro-foci of PTC

**Fig. 8:**
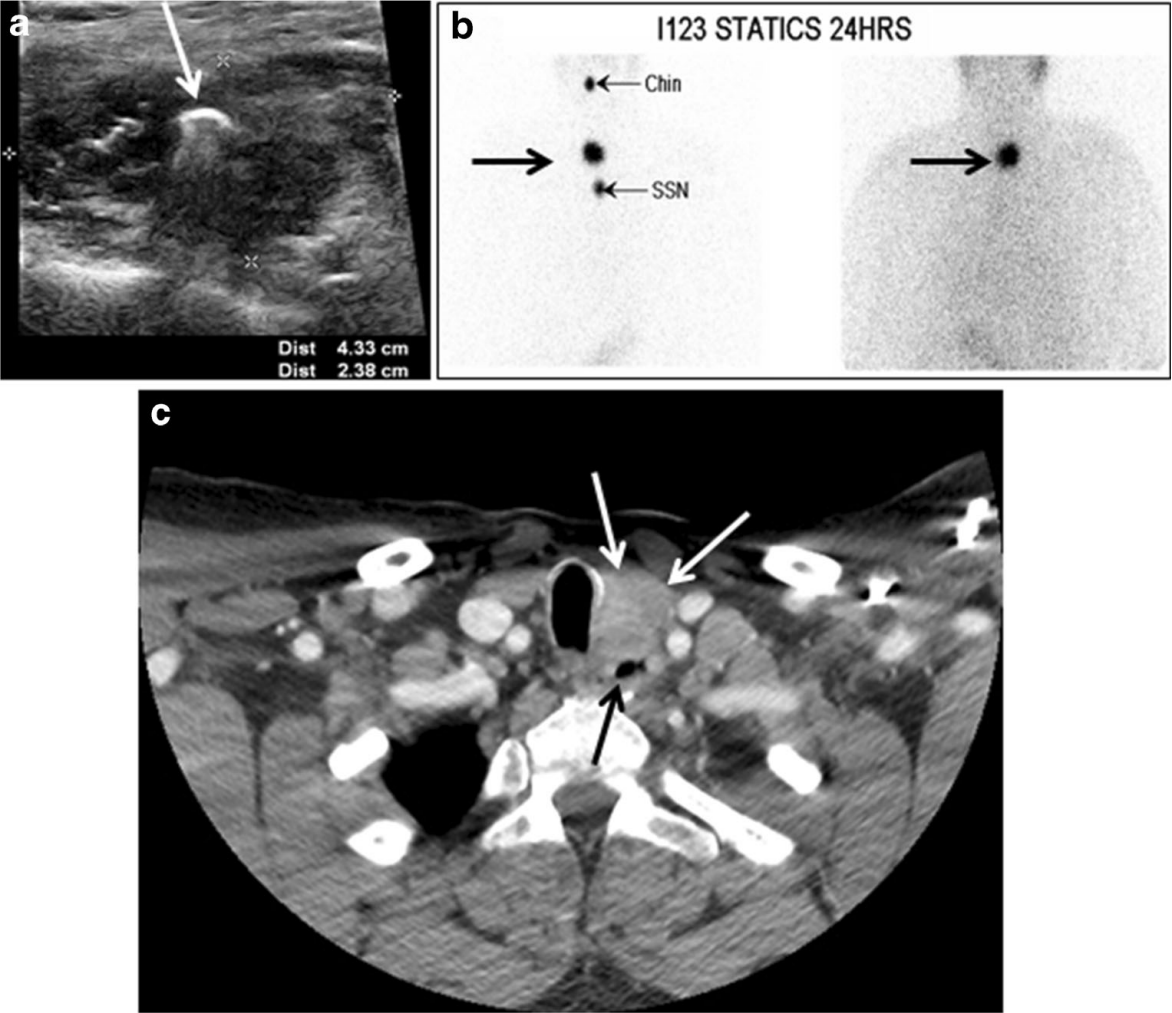
A 48-year-old male patient post total thyroidectomy with PTC recurrence. **a** Transverse greyscale ultrasound of the neck demonstrates a left thyroid bed heterogeneous, predominantly hypoechoic irregular lesion with calcifications (*white arrow*). **b** A spot image of iodine 123 total body scan of the neck demonstrate a focus of abnormal radiotracer uptake at the left thyroid bed (*Black arrows*) between the annotated markers. **c** Enhanced axial CT scan of the neck demonstrates an enhancing large left thyroid bed mass (*white arrow*) with no calcifications. The lesion exerts a mass effect on the oesophagus (*black arrow*) and is inseparable from the trachea

**Fig. 9:**
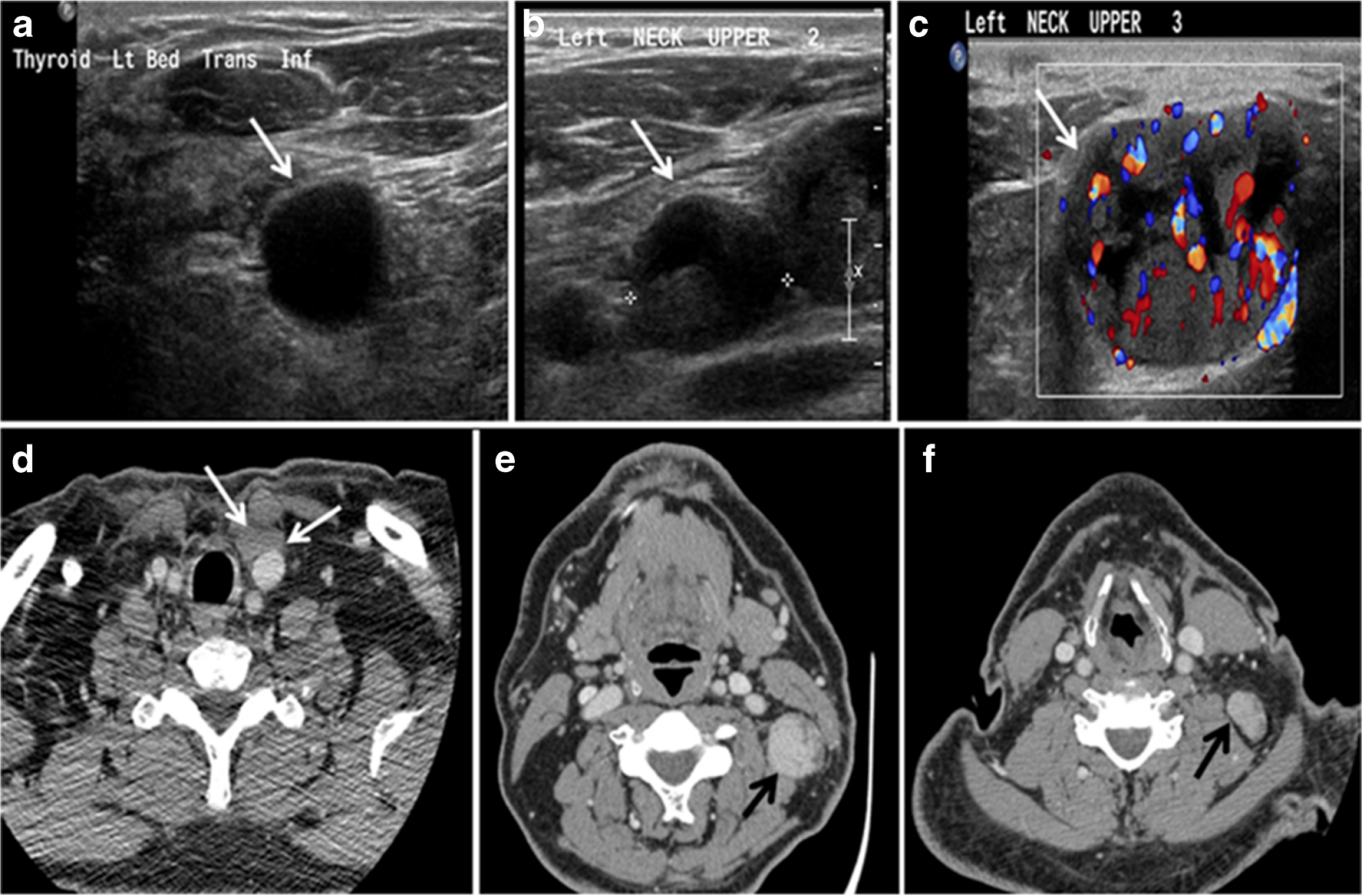
A 58-year-old male patient with persistence PTC at thyroid bed with hypervascular nodal metastasis. **a**–**c** Transverse greyscale and colour Doppler neck ultrasound demonstrate hypoehoic soft tissue in the left thyroid bed (*white arrow* in **a**). There are a heterogeneous enlarged lymph nodes at level 2 and 3 with markedly increased vascularity (white arrow in **b** and **c**). **d**–**f** Enhanced axial CT images of the neck demonstrate a 2.7 × 1.4 cm hypodense soft tissue lesion anterior to the left carotid sheath (*white arrow*). There are left-sided enhancing abnormal and enlarged lymph nodes at cervical level 2 and 3 (*black arrows*)

The likelihood of positive anatomic imaging is greater when serum Tg is >10 ng/mL [16]. A diagnostic CT scan adds additional value to neck US in detecting central compartment macro-metastases in the mediastinum and retro-tracheal area [34–36]. According to the recent American Thyroid Association guidelines [16], an upper chest and neck CT scan with IV contrast should be obtained when: 1) neck US is inadequate in visualizing possible local nodal disease (high Tg, negative neck US, and RAI imaging); 2) US is not able to delineate the disease completely, as in the case of bulky recurrent nodal disease; or 3) evaluation of possible recurrent invasive disease is needed (Figs. 7, 8 and 9). CT scans are also the most sensitive diagnostic tool for the detection of pulmonary micro-metastases. Many of the neck US features that are considered as suggestive signs of disease recurrence are also applicable to CT examination. These signs might include sizable rounded nodules in the thyroid bed, fine calcifications, or cystic change [37].

In cases of elevated thyroglobulin with negative neck US and iodine whole body scintigraphy (WBS), fluorodeoxyglucose (FDG) positron emission tomography (PET) is the next modality of choice. Dedifferentiated thyroid carcinoma usually has avid FDG-PET uptake and a negative radioiodine scan, typically does not respond to RAI therapy, and has a poorer prognosis [2]. There is not yet consensus in the research literature on whether cross-sectional imaging (CT or MRI) or an 18FDG-PET/CT scan should be performed as the first-line imaging modality for such patients. Enhanced CT scan was thought to be more sensitive for detection of lymph node metastases. Nonetheless, scans using modern PET/CT equipment are as reliable as a proper routine staging CT scan. Many lesions can be found on 18FDG-PET/CT scanning despite the lack of IV contrast injection. However, differentiation between local recurrence versus lymph node metastases and detection of direct involvement of the aerodigestive axis or vascular structures are not technically possible in the absence of IV contrast administration. For these reasons, 18FDG-PET/CT utilizing contrast administration should be considered for most patients with extensive disease [16, 38–40].

### Metastasis to the thyroid

**Fig. 10:**
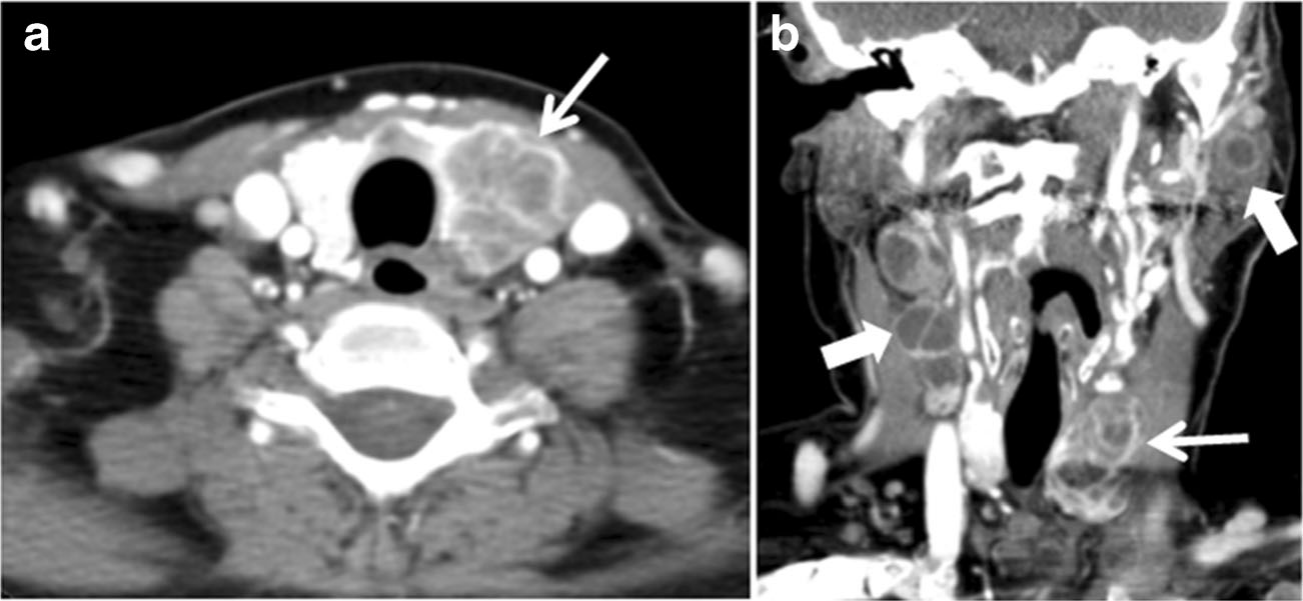
Metastatic squamous cell carcinoma of unknown origin in a 42-year-old female patient. **a**, **b** Axial and coronal enhanced neck CT scan demonstrates infiltrative hypodense left thyroid lobe lesions (*white arrows*). There are multiple necrotic cervical nodal metastases (*white block arrows*)

Metastasis to the thyroid is rare and represents 5.5 % of biopsied thyroid malignancies. It is commonly found with cancers originating from the breast, renal cell, lung, melanoma, and colon. Direct invasion from adjacent structures such as the pharynx, larynx, trachea, or oesophagus has been reported (Fig. 10). Metastatic disease has a non-specific appearance [1, 2, 20, 21].

Presence of ITNs in patients with another known malignancy is a common clinical problem with controversial management guidelines. Wilhelm et al. [41] followed 41 patients with a known extra-thyroid malignancy and ITNs; 35 of them met the criterion for biopsy (nodule ≥ 1 cm). Pathology revealed four papillary thyroid cancers and five micropapillary thyroid cancers. Only two metastatic cancers were detected. Clinical history (history of radiation, age, endocrine syndromes), TSH, nodule size, and sonographic features are important to determine which nodule(s) should be followed or biopsied. However, existing guidelines do not specifically address how to approach ITNs detected on CT scans in such a specific patient population [41].

### Thyroid lymphoma

**Fig. 11:**
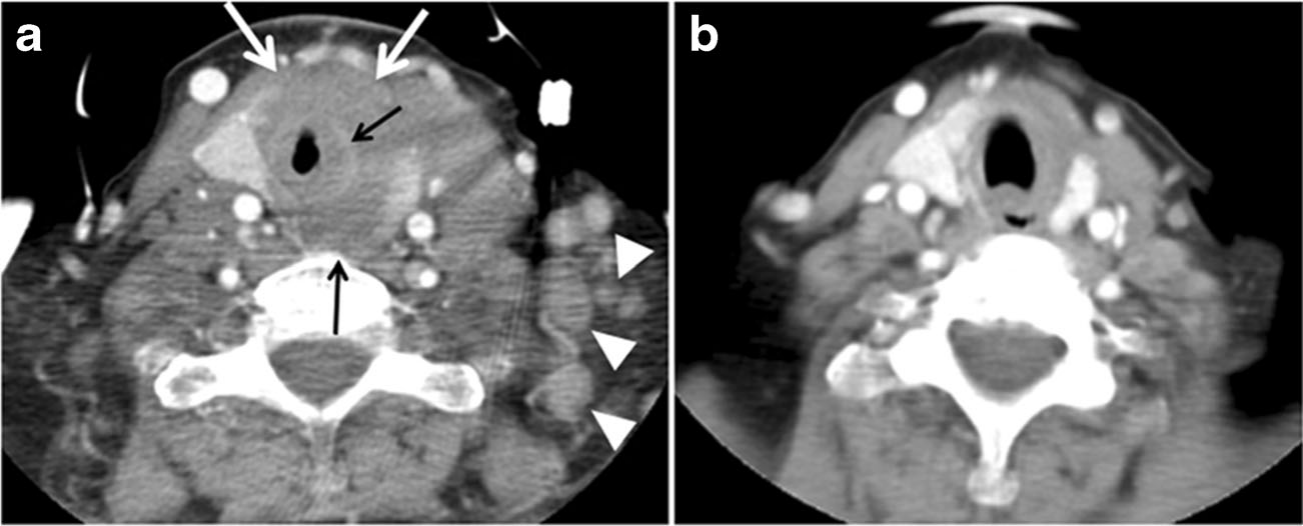
Thyroid non-Hodgkin’s large B-cell lymphoma in a 66-year-old female patient. **a** Axial enhanced neck CT scan demonstrates left thyroid lobe and isthmus homogeneously hypodense and minimally enhancing mass (*white arrows*). This lesion invades the prevertebral muscles (*black arrows*). Note the multiple enlarged level V lymph nodes (*white arrowheads*). **b** Post-treatment image shows significant reduction in size and mass effect of the left thyroid infiltrative mass, with almost complete resolution of the left cervical lymphadenopathy

**Fig. 12:**
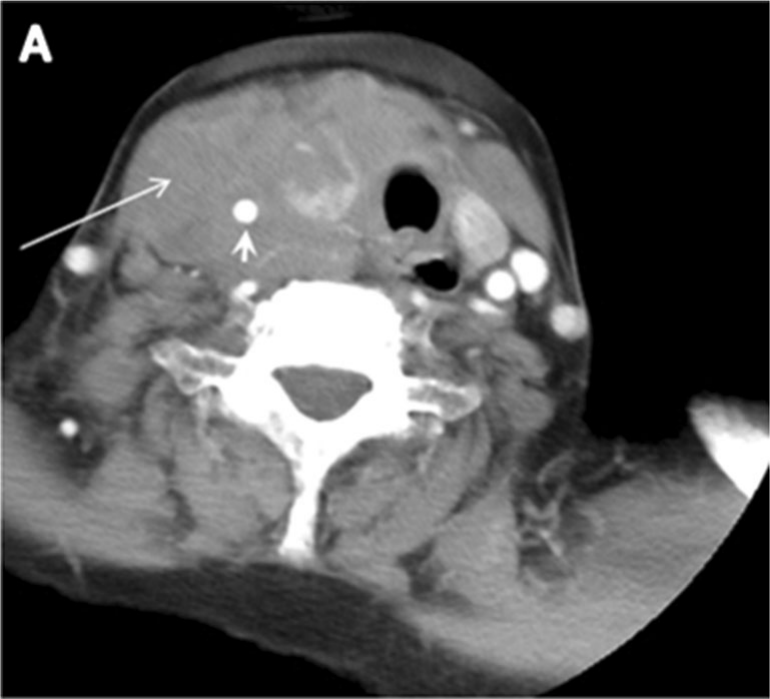
Diffuse thyroid large B-cell lymphoma in 79-year-old female patient. **a** Axial enhanced neck CT scan demonstrates a homogeneously hypodense and minimally enhancing large right thyroid solid mass (*long white arrow*) extending into the thyroid isthmus. It is encasing the right carotid artery (*short white arrow*) and displacing of the trachea and oesophagus to the left side

Thyroid lymphoma represents about 5 % of thyroid malignancies. Non-Hodgkin’s lymphoma is the most common type and can be secondary to generalized lymphoma or a primary tumour. Primary thyroid lymphoma usually pre-exists with Hashimoto’s thyroiditis. On CT scans with and without contrast, lymphomas tend to have low attenuation values. Thyroid lymphomas have variable appearance and mostly manifest as a solitary mass (80 %). They may also manifest as multiple nodules (15 % to 20 %) or as a bulky mass replacing the entire gland with extra-thyroid extension (Figs. 11 and 12). The presence of cervical lymphadenopathy supports such a diagnosis. Although it is uncommon, tumour necrosis has been reported [1, 21].

## Goiter

**Fig. 13:**
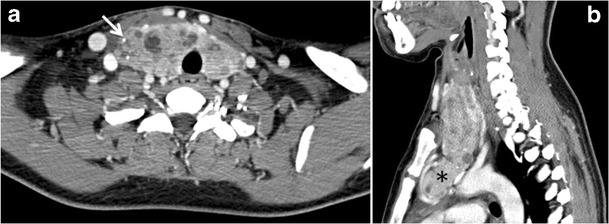
A 27-year-old female patient known to have goiter. **a**, **b** Axial and sagittal enhanced CT scan images of the neck demonstrate a heterogeneously enhancing, enlarged thyroid gland with scattered calcifications (*white arrow*), cystic changes, and substantial retro-sternal extension (*black asterisks*). No lymphadenopathy or substantial airway narrowing

**Fig. 14:**
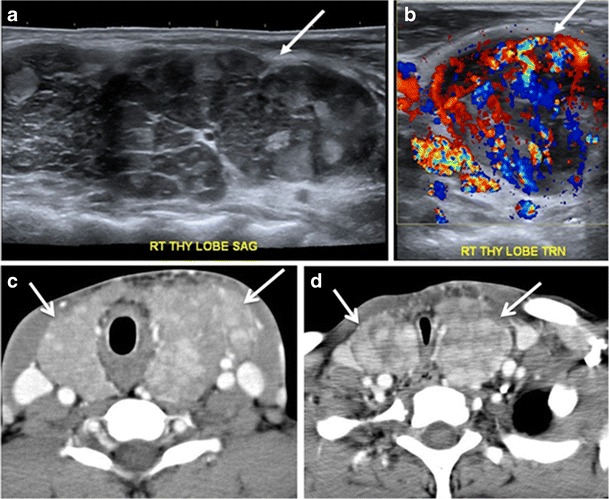
A 19-year-old male patient known to have multi-nodular goiter and FNA, showing underlying Hashimoto’s thyroiditis. **a**, **b** Sagittal and transverse greyscale and colour Doppler ultrasound of the neck demonstrate a hypoechoic enlarged right thyroid lobe with small hyperechoic regenerative nodules and marked hypervascularity (*white arrows*). **c**, **d** Enhanced axial CT scan images of the neck demonstrate a heterogeneously enhancing and enlarged thyroid gland, left more than right lobe, and the trachea is markedly narrowed

**Fig. 15:**
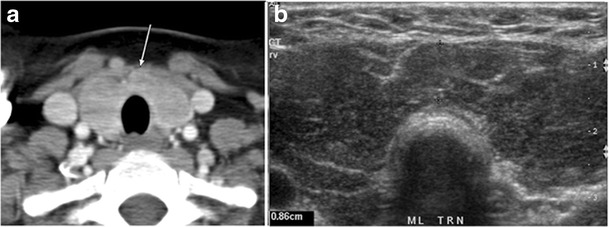
A 33-year-old female patient who presented with neck swelling and pain and was later diagnosed with Hashimoto’s thyroiditis. **a** Axial enhanced CT scan of the neck demonstrates minimal diffuse enlargement of the thyroid gland, especially the isthmus (*white arrow*). **b** Transverse greyscale ultrasound of the neck demonstrates heterogeneously enlarged thyroid and thickened isthmus, measuring 8.6 mm

A goiter is an abnormal thyroid gland proliferation that manifests as multi-nodular, uni-nodular, or non-nodular diffuse glandular enlargement. A goiter is formed of solid matrix, colloid cysts, blood products, calcification, and fibrosis, and this heterogeneity may lead to variable appearances on a CT scan (Figs. 13, 14 and 15) [2]. US is more sensitive in evaluating thyroid nodules within a goiter; however, a symptomatic goiter may require surgical treatment with total thyroidectomy [42], and in this case CT plays an additional role in preoperative evaluation. Specific aspects for examination on a CT scan during the preoperative evaluation for goiter include extension, mass effect, and suspicious features of malignancy [2, 43].

Malignancy can coexist within the goiter and a CT scan may give a clue if there are abnormal cervical lymph nodes and/or signs of invasion. Retrosternal extension (Fig. 15) could affect the surgical approach, as a lower extent may require a partial or total sternotomy to facilitate complete resection [43]. Therefore, the distance of the retrosternal extent from the sternal notch should be measured on a sagittal image.

The interpreting radiologist should describe the mass effect, detailing its degree and direction of displacement of central structures, including the trachea, oesophagus, larynx, and pharynx. Attention should be directed to the upper extent of the goiter and structures immediately surrounding the thyroid gland, including the neuro-vascular structures, retropharyngeal space, and pre-vertebral space. The reporting radiologist should evaluate the vocal cords for symmetry and signs of vocal cord palsy [2, 43].

## Inflammatory lesions

**Fig. 16:**
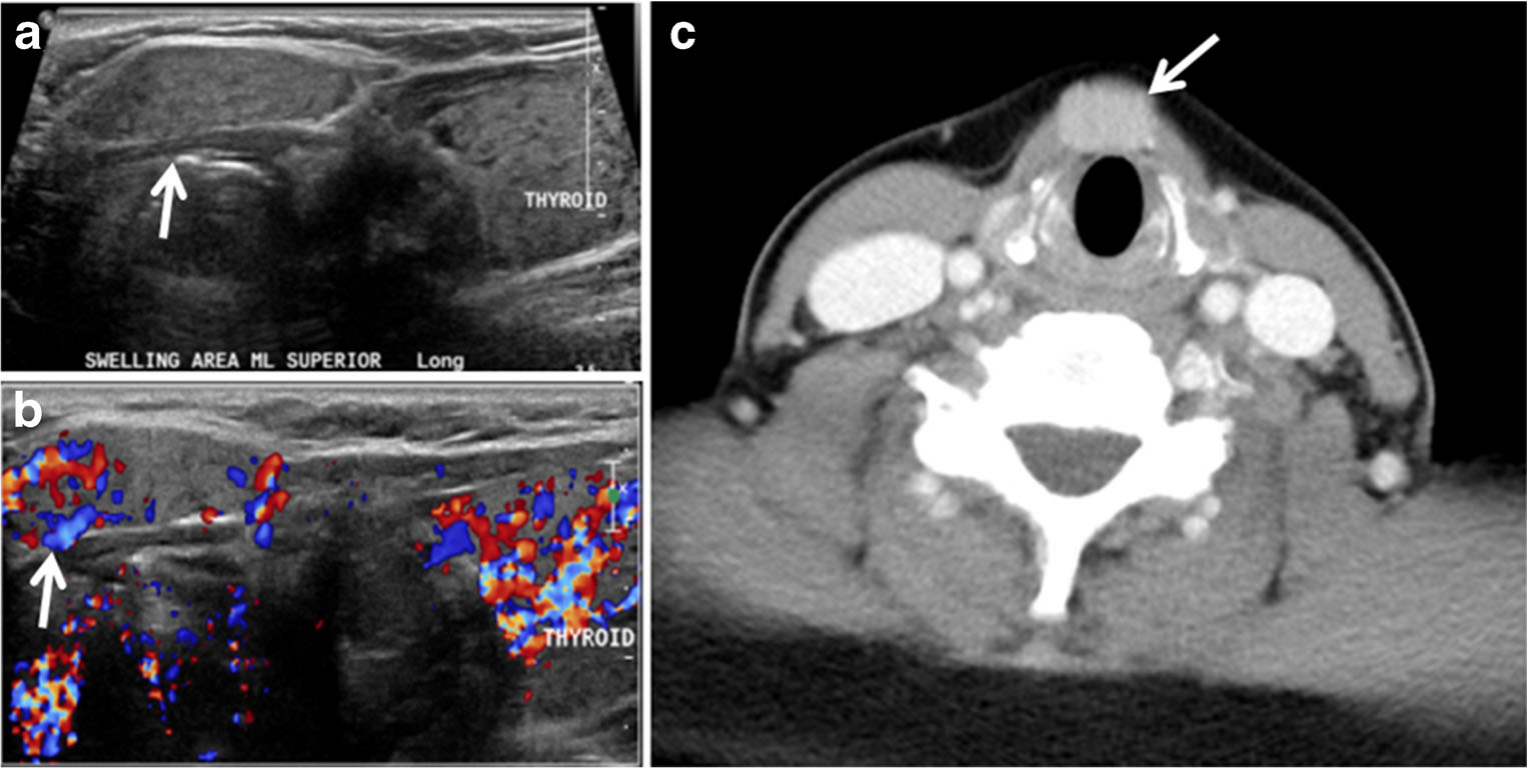
Midline ectopic thyroid with Hashimoto’s thyroiditis in a 49-year-old female patient. **a** Transverse greyscale ultrasound shows a 1.6 × 0.8 cm solid, well-defined, heterogeneous area (*white arrow*) in the midline, superior to the thyroid gland. It is iso-echogenic to the thyroid gland with no definite connection to the thyroid gland. **b** Transverse colour Doppler ultrasound shows significant increase in vascularity. **c** Axial enhanced neck CT scan at the level of thyroid cartilage demonstrates midline infrahyoid hyperdense soft tissue mass (*white arrow*) embedded within the strap muscle

Inflammatory thyroid disorders include acute infectious thyroiditis, Hashimoto’s thyroiditis, Riedel’s thyroiditis, and granulomatous thyroiditis (de Quervain’s). Hashimoto’s thyroiditis is associated with an increased risk of lymphoma and papillary thyroid carcinoma. The CT scan findings of thyroiditis are nonspecific and variable (Figs. 14, 15 and 16) [1]. The thyroid gland has a very high iodine concentration, resulting in high CT attenuation (80–100 Hounsfield Units). The presence of thyroiditis can be suggested by a diffusely enlarged and hypo-attenuating (around 45 Hounsfield Units) thyroid gland. This is probably due to follicular cell destruction and reduced thyroid iodine concentration. Marked homogenous enhancement is typically expected. Therefore, moderate thyroid enhancement in a case of thyroiditis suggests a diffuse inflammatory process. It is essential to clinically correlate this with a thyroid function test and serum autoantibody levels [1, 44].

## Ectopic tissue/gland

During embryogenesis, the bi-lobed thyroid migrates inferiorly from the foramen cecum of the tongue to the lower neck. Initially, the thyroid primordium passes anterior to the primordial hyoid bone, before it loops posteriorly and inferiorly to the hyoid bone. Then it continues its descent into the infra-hyoid portion of the neck, anterior to the trachea, thyroid cartilage, and thyroid membrane. Any thyroid residual along the descent course may lead to the development of ectopic thyroid glands. Thyroid carcinomas, thyroiditis, and goiter may develop within any ectopic thyroid tissue [1, 45–47].

Thyroid scanning with technetium-99 m (Tc99m) plays an important role in detecting orthotopic and ectopic thyroid tissue. Both CT scans and US can help detect ectopic tissue when a lesion demonstrates imaging and enhancement characteristics of thyroid tissue. The absence of normally sited thyroid gland in US and CT scans also supports the diagnosis. In addition, US can guide FNA for cytological confirmation of a thyroid lesion [46]. Ectopic thyroid tissue appears as a well-circumscribed, homogeneous, highly attenuating mass relative to adjacent muscles. Normally, it enhances avidly following the administration of iodinated contrast [1, 45, 47].

**Fig. 17:**
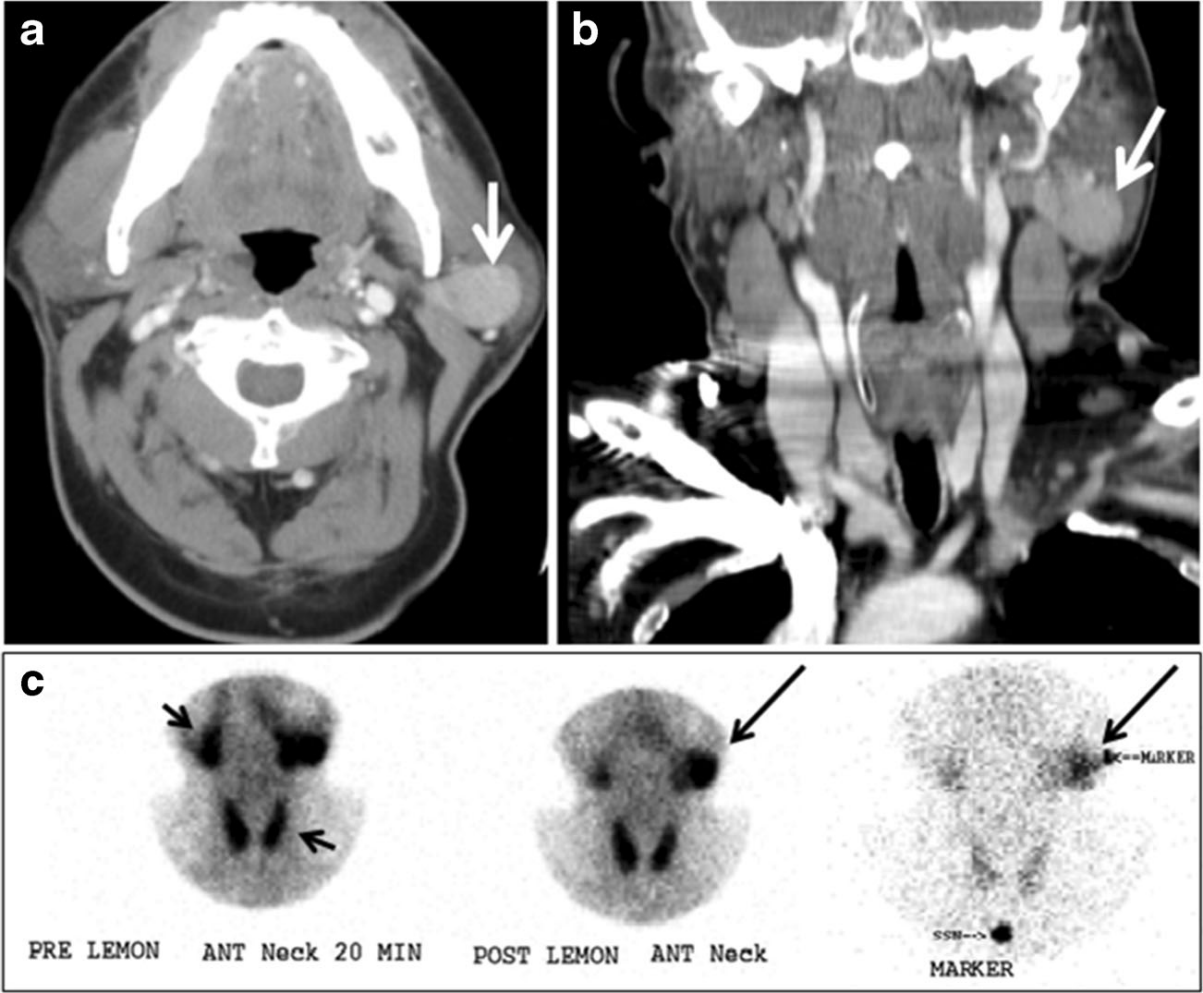
Ectopic thyroid on the left parotid gland with a palpable left parotid mass in a 69-year-old male patient. **a**, **b** Axial and coronal enhanced neck CT scan demonstrates well-defined homogeneous enhancing mass (*white arrows*) within the left parotid gland with preserved surrounding fat planes. It also shows a normal thyroid in normal position in the lower neck. **c** Image taken 20 minutes after 5 mCi injected Tc99m-Pertechnetate shows normal thyroid uptake of tracer and physiological uptake in the salivary glands (*short black arrow*). There is a distinct focus of abnormal tracer accumulation in the left parotid/submandibular region. Patient was given lemon juice with evident normal washout from the salivary glands and relative retention by this abnormal focus (*long black arrow*)

**Fig. 18:**
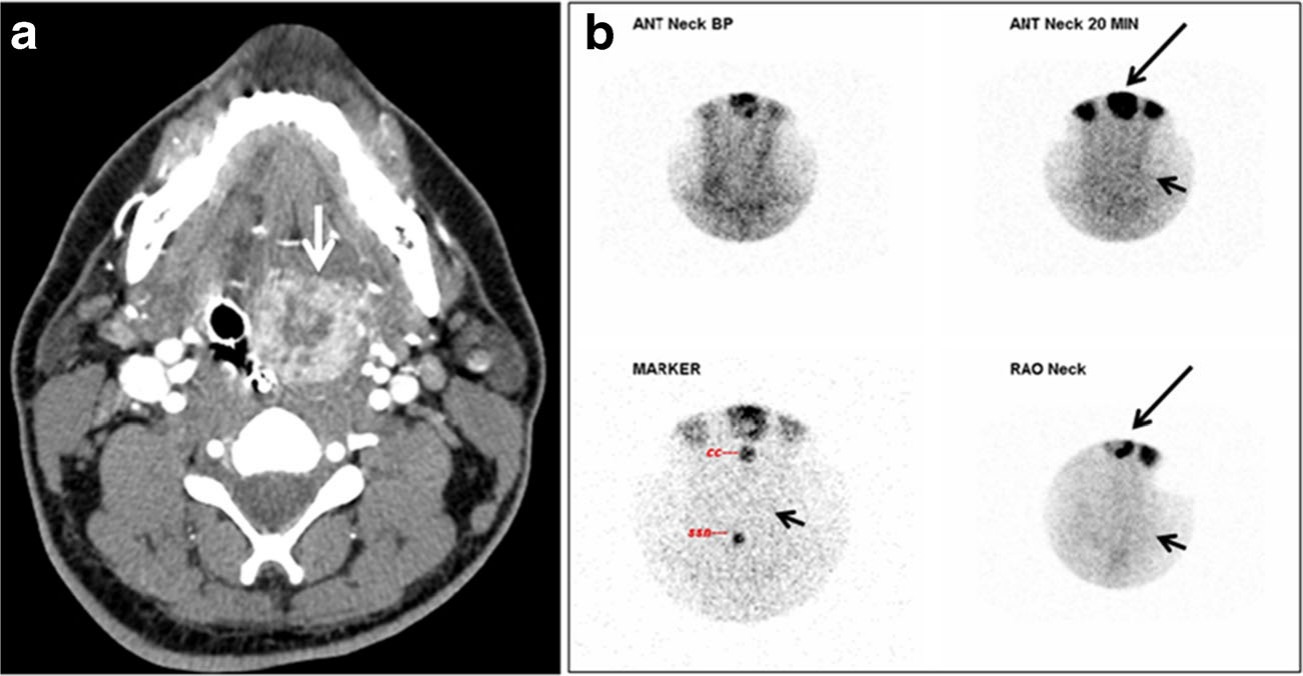
Lingular thyroid in a 33-year-old male who presented with oropharyngeal bleeding. **a** Axial enhanced neck CT scan at the level of mandible demonstrates a 3 × 3 × 3.4 cm round, partly well-delineated, heterogeneously enhancing lesion (*white arrow*). It is predominantly on the left side of the oropharynx and to some extent at the mid part of the base of the tongue. Thyroid gland was normal (not shown). **b** Image of the anterior face and neck taken 20 minutes after Tc99m-Pertechnetate injection shows absent thyroid radiotracer uptake in normal thyroid anatomical location (*black short arrows*). There is an area of increased uptake (*long black arrows*) corresponding to posterior tongue mass identified on CT scan

Ectopic thyroid tissue may be detected in the tongue near the foramen cecum (90 %) and along the midline between the thyroid isthmus and posterior tongue, lateral neck, mediastinum, and oral cavity. The most frequent location is the base of the tongue (Figs. 16, 17 and 18). In 70 % of cases, the ectopic thyroid is the only functional thyroid tissue present in the body (Fig. 18) [1, 45, 46].

Ectopic thyroid tissue lateral to the orthotopic midline location is rare [46, 48, 49]. The exact anatomical definition of this rare entity is debated in the literature [48, 50]. To avoid confusion, some authors define a lateral neck ectopic thyroid as any thyroid tissue superficial to the strap muscles with no midline continuity [45]. The majority of lateral thyroid ectopia cases have been reported as lesions closely related to the strap muscles [48, 49]. There are few reported cases of ectopic lateral thyroid tissue in the submandibular region [46, 51], jugulodigastric region [52], or within the parotid gland substance (Fig. 17) [53].

The origin of lateral ectopic thyroid tissue is not fully understood. Although this is controversial, some authors suggest that it might have originated from lateral thyroid anlagen (ultimobranchial bodies) that failed to fuse with the median anlage during caudal migration [46, 48].

**Fig. 19:**
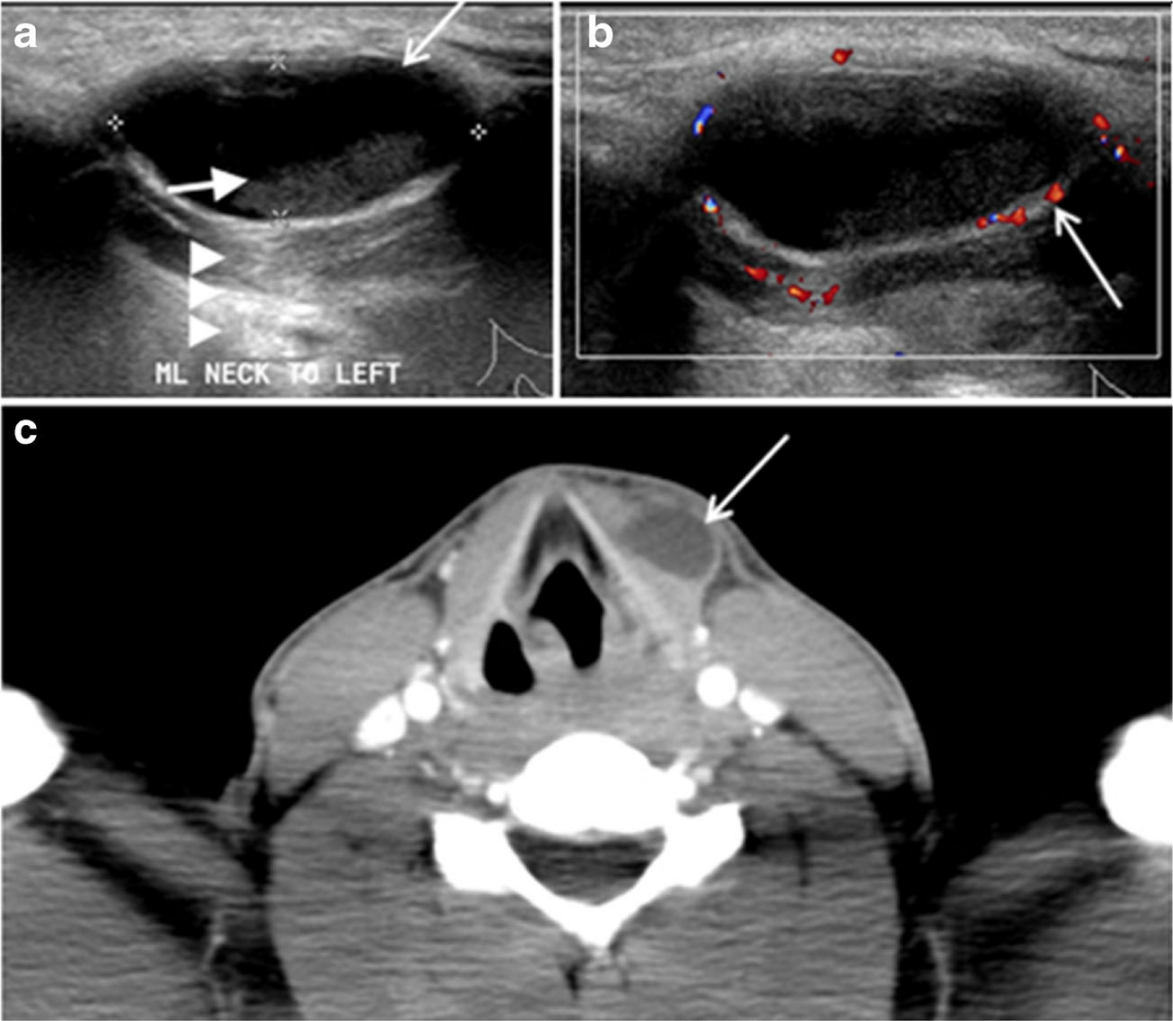
Long-standing infected thyroglossal duct cyst in a 29-year-old male patient. **a** Transverse greyscale ultrasound at midline, just above the level of the thyroid gland, shows an oval cystic lesion with internal echoes (*white arrows*) and posterior enhancement (*arrowheads*). **b** Transverse colour Doppler ultrasound shows surrounding peripheral flow (*white arrow*). **c** Axial enhanced neck CT scan at the level of the thyroid cartilage demonstrates a slightly off-midline, well-defined, homogeneous cystic lesion embedded in the left strap muscle with peripheral enhancement (*white arrow*). It shows no calcification or internal enhancement. Thyroid gland was normal (not shown)

**Fig. 20:**
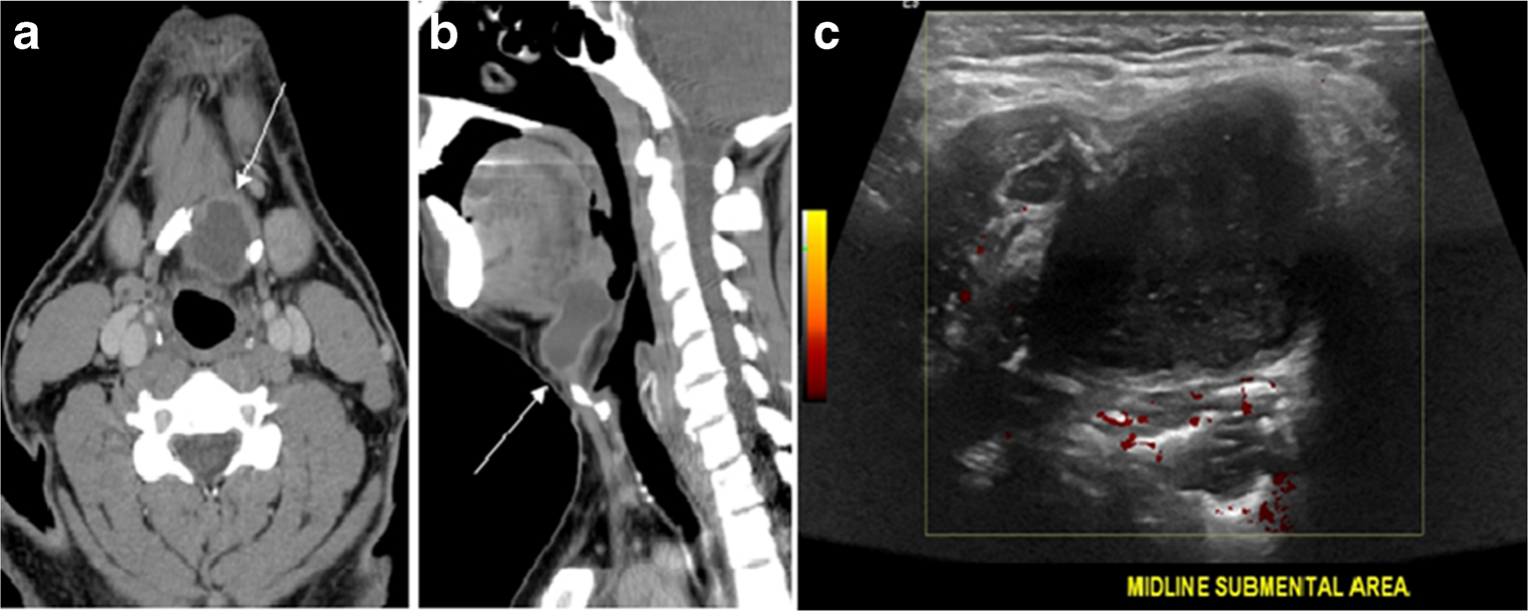
Recurrent/residual thyroglossal duct cyst in a 39-year-old male patient. The first resection of thyroglossal duct cyst showed histopathology evidence of Hurthle cell type thyroid cancer. However, the second resection showed signs of chronic inflammation, with no malignant cells. **a**, **b** Enhanced axial and sagittal neck CT scans demonstrate a unilocular cystic lesion arising from the tongue base and extending through the partially resected hyoid bone. This cystic lesion has peripheral enhancing wall, which becomes more thick over its inferior aspect associated with surrounding fat stranding at the surgical site (*white arrow*). There are no internal septations, nodules or masses, or calcifications. **c** Transverse view of power Doppler ultrasound at the submental area demonstrates cystic lesion and internal debris with no detected internal vascularity

**Fig. 21:**
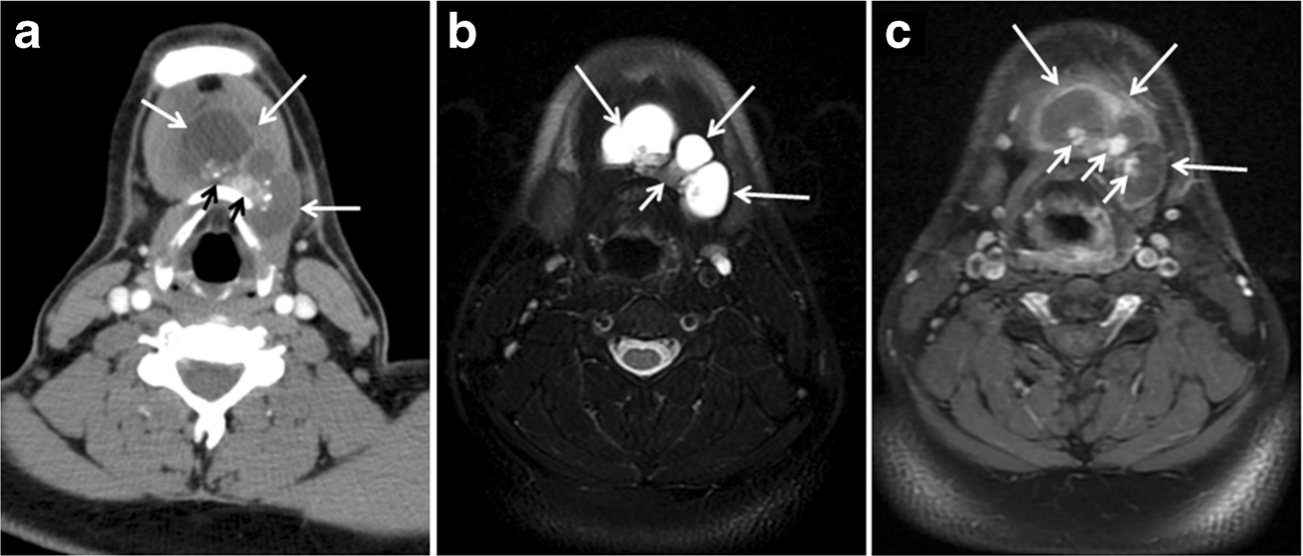
Papillary thyroid carcinoma arising from a thyroglossal duct cyst in a 28-year-old male. **a** Axial enhanced CT scan shows a large complex cystic lesion (*white arrows*) adherent to the anterior aspect of the hyoid bone. It has an enhancing mural solid nodules and calcifications (*black arrows*). There is no cervical lymphadenopathy. **b** Axial short tau inversion recovery (STIR) MRI image near the same level shows complex lesion of high signal intensity (*long white arrows*) with solid mural nodules (*short white arrow*). **c** Axial fat saturated T1 MRI image post contrast administration shows the complex cystic lesion with thick enhancing wall (*long white arrows*) and enhancing mural nodules (*short white arrows*)

A thyroglossal duct cyst (TDC) is a duct remnant between the foramen cecum and thyroid isthmus. Most TDCs are located below the hyoid bone and in the midline. The more caudal the cyst, the more likely it will be off midline within 2 cm (Fig. 19 and 20). On a CT scan, a TDC appears as a well-circumscribed area of fluid attenuation with thin walls. The cyst wall can become thick with an enhancing rim indicative of current or previous infection. These cysts maybe complicated by haemorrhage, infection, or malignancy. Therefore, their US and CT scan appearance may vary based on their content. Nodular enhancement within a TDC should initiate further workup to exclude malignancy (Fig. 21) [1, 45]. US-guided FNA of these suspicious nodular areas is considered an appropriate next diagnostic step, taking into consideration the high rate of false negative results [54].

## Intra-thyroid parathyroid adenoma

**Fig. 22:**
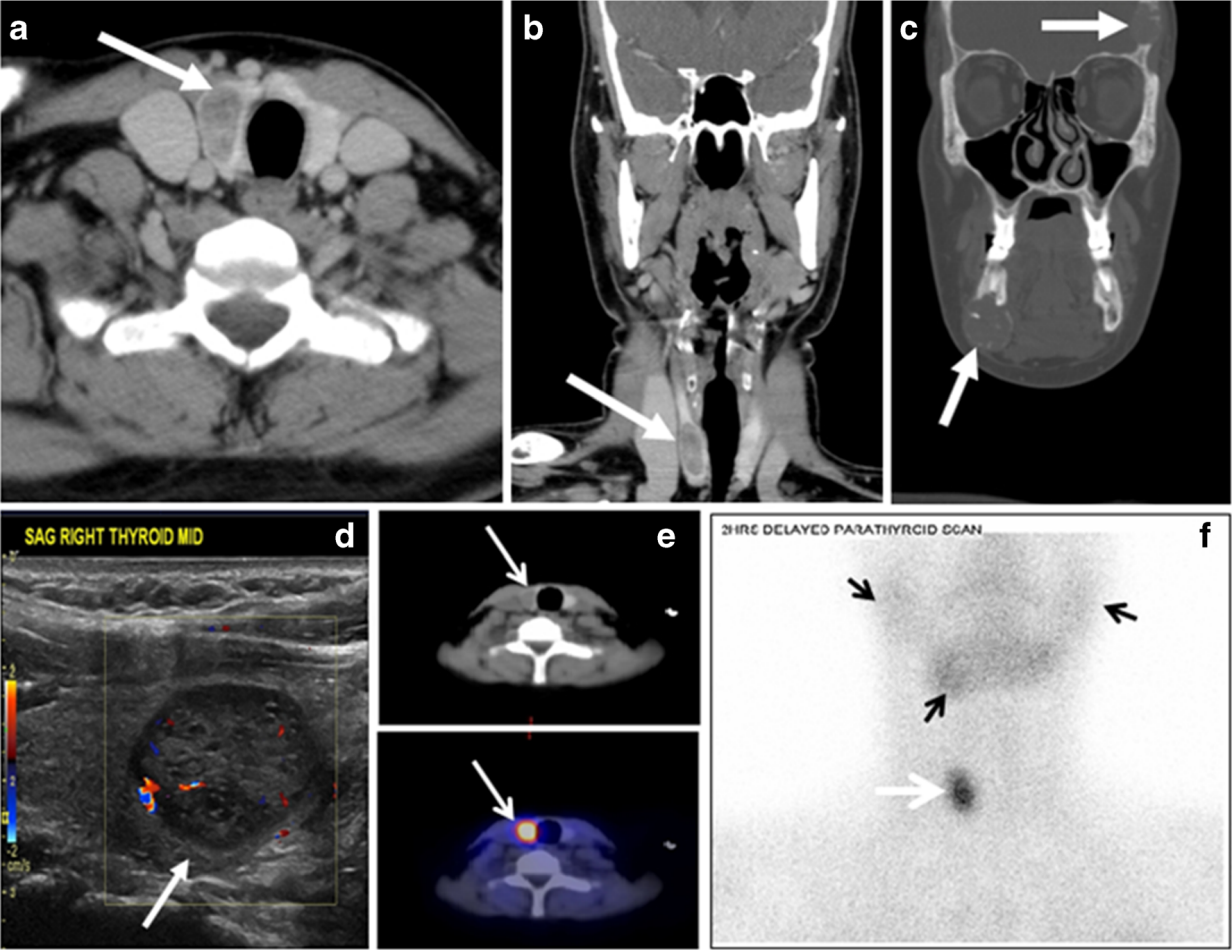
A 26-year-old male patient with elevated serum parathyroid hormones and calcium secondary to intra-thyroid parathyroid adenoma. **a**, **b** Enhanced axial and coronal CT scan of the neck demonstrate a well-defined hypodense right thyroid nodule (*white arrows*). **c** Bone window coronal CT scan shows lytic expansile lesions at the right mandible and left frontal bone (*white arrows*). **d** Transverse colour Doppler ultrasound of the neck demonstrates a well-defined, heterogonous, predominantly hypoechoic right thyroid nodule measuring 2.7 cm, with mild increased vascularity and no internal micro-calcifications (*white arrow*). **e**, **f** Delayed anterior planar and fused SPECT/CT parathyroid Sestamibi scan at 2 hours demonstrate persistent focal activity in the right thyroid lobe (*white arrows*). Note the scattered mandibular/maxillary uptakes in planar image representing the known brown tumours

Parathyroid adenoma (PA) is the most common cause of primary hyperparathyroidism. Ectopic parathyroid adenoma is rare. The third and fourth pharyngeal pouches represent the embryological origin of the parathyroid tissues, and ectopic parathyroid adenoma can ultimately develop anywhere along their migration course. In a large retrospective study of patients with primary hyperparathyroidism, PA was detected in the intra-thyroid location in 0.7 % of cases. In another retrospective analysis of 202 patients with ectopic PA, intra-thyroidal location was found in 18 % of the cases. Intra-thyroid parathyroid adenomas mimic thyroid nodules in CT scans and may even show uptake on a thyroid iodine scan. Correlation with laboratory workup, including measurement of serum parathyroid hormone and calcium level, is required. In addition, the evaluating radiologist should search for radiological manifestations of hyperparathyroidism, such as osteopenia, bone resorption, and brown tumours (Fig. 22) [55, 56].

In the case of inconclusive Tc99m Sestamibi and neck US imaging, FNA biopsy with FNA-iPTH (intact parathyroid hormone) measurement can provide simultaneous biochemical and cytological evidence. Elevated FNA-iPTH measurement, as compared to serum iPTH, is considered positive and diagnostic of parathyroid adenoma [57, 58].

## Conclusion

Thyroid disorders are common and tend to have non-specific appearances on CT scans. Commonly encountered findings when evaluating a CT scan of the neck include thyroid nodules, glandular enlargement, and calcifications.

Management of ITNs depends on several factors including nodule size, patient’s age, overall health status, and the presence or absence of suspicious features such as lymphadenopathy and/or invasion of adjacent structures.

A CT scan provides additional important information regarding the local extension of cancer or presence of mass effect, and is useful in evaluating recurrent disease. Furthermore, CT examination plays a crucial role in preoperative evaluation and preoperative surgical planning for patients with symptomatic goiter.

## Abbreviations

ITN: Incidental thyroid nodule
CT: Computed tomography
US: Ultrasound
FNA: Fine needle aspiration
IV: Intravenous
MRI: Magnetic resonance imaging
PTC: Papillary thyroid carcinomas
ACR: American College of Radiology
TSH: Thyroid stimulating hormone
DTC: Differentiated thyroid carcinomas
RAI: Radioactive iodine
Tg: Thyroglobulin
TgAb: Antithyroglobulin
WBS: Whole body scintigraphy
FDG: Fluorodeoxyglucose
PET: Positron emission tomography
Tc99m: Technetium-99 m
TDC: Thyroglossal duct cyst
PA: Parathyroid adenoma
iPTH: Intact parathyroid hormone
